# Palmatine from Unexplored *Rutidea parviflora* Showed Cytotoxicity and Induction of Apoptosis in Human Ovarian Cancer Cells †

**DOI:** 10.3390/toxins11040237

**Published:** 2019-04-25

**Authors:** Okiemute Rosa Johnson-Ajinwo, Alan Richardson, Wen-Wu Li

**Affiliations:** 1 Guy Hilton Research Centre, School of Pharmacy and Bioengineering, Keele University, Stoke-on-Trent ST4 7QB, UK; okiemute_2002@yahoo.co.uk (O.R.J.-A.); a.richardson1@keele.ac.uk (A.R.); 2 Faculty of Pharmaceutical Sciences, University of Port Harcourt, Port Harcourt, PMB 5323, Nigeria

**Keywords:** Ovarian cancer, *Rutidea parviflora*, Palmatine, Apoptosis

## Abstract

Ovarian cancer ranks amongst the deadliest cancers in the gynaecological category of cancers. This research work aims to evaluate in vitro anti-ovarian cancer activities and identify phytochemical constituents of a rarely explored plant species—*Rutidea parviflora* DC. The aqueous and organic extracts of the plant were evaluated for cytotoxicity using sulforhodamine B assay in four ovarian cancer cell lines and an immortalized human ovarian epithelial (HOE) cell line. The bioactive compounds were isolated and characterized by gas/liquid chromatography mass spectrometry and nuclear magnetic resonance spectroscopy. Caspase 3/7 activity assay, western blotting and flow cytometry were carried out to assess apoptotic effects of active compounds. The extracts/fractions of *R. parviflora* showed promising anti-ovarian cancer activities in ovarian cancer cell lines. A principal cytotoxic alkaloid was identified as palmatine whose IC_50_ was determined as 5.5–7.9 µM. Palmatine was relatively selective towards cancer cells as it was less cytotoxic toward HOE cells, also demonstrating interestingly absence of cross-resistance in cisplatin-resistant A2780 cells. Palmatine further induced apoptosis by increasing caspase 3/7 activity, poly-ADP-ribose polymerase cleavage, and annexin V and propidium iodide staining in OVCAR-4 cancer cells. Our studies warranted further investigation of palmatine and *R. parviflora* extracts in preclinical models of ovarian cancer.

## 1. Introduction

Ovarian cancer is a significant and global threat to life in women. American cancer society estimated that 22,240 of new ovarian cancer diagnosed and 14,070 ovarian cancer deaths are projected to occur in the United States in 2018 [1]. It is ranked 5th most common cause of death among women in the UK. There were 7270 new cases of ovarian cancer in 2015 and 4227 cases of ovarian cancer-related deaths in 2016 in the UK. The ten year survival rate (2010–2011) remains just 35% [2]. Epithelial ovarian cancer can be subdivided into at least four major histological subtypes: serous, endometrioid, clear cell and mucinous carcinoma [3]. High-grade serous ovarian cancer (HGSOC), the most aggressive subtype, is responsible for 70–80% of all ovarian cancer deaths; the overall survival rate has not changed significantly for several decades [4,5]. Ovarian cancer is typically diagnosed at a late stage and no effective screening strategy exists. The current standard treatment for ovarian cancer entails surgery aimed at removing most of the cancerous cells, followed by the administration of chemotherapeutics, often resulting in multiyear survival [4]. However, use of chemotherapy introduces drug resistance and consequently subsequent relapse can lead to the death of the cancer patients [6,7]. For example, platinum-based drugs are initially effective against HGSOC, but recurrent tumors resistant to these agents have developed later on [8]. Recently, three novel poly-ADP-ribose polymerase (PARP) inhibitors such as olaparib, rucaparib, and niraparib have been approved by the US Food and Drug Administration and European Medicines Agency for the treatment of ovarian cancer caused by the alteration of DNA damage repair pathways [9]. Despite of these significant achievements, investigation into the discovery of new drugs that may offer wider therapeutic benefits by overcoming resistance mechanisms and drug toxicity in ovarian cancer therapy is still an unmet need in the light of the ovarian cancer menace.

The use of plants in the treatment of cancer is not new because there is documented evidence of the use of medicinal plants for the treatment of cancer [10,11,12]. A number of plant-derived or semi-synthetic anti-ovarian cancer drugs including paclitaxel, etoposide and topotecan have been approved and widely used in clinic [13,14]. Previously, we identified cytotoxic cyclotides from a Chinese medicinal plant [15], a cytotoxic indolizine alkaloid, securinine [16,17], and three cytotoxic bisbenzylisoquinoline alkaloids, cycleanine [18,19,20], isochondodendrine and 2’-norcocsuline [21,22] in Nigerian medicinal plants. Semi-synthetic cycleanine [23] and thymoquinone [24] analogues were further prepared and evaluated for their in vitro anti-ovarian cancer activities. In continuation of our search for novel anti-ovarian cancer compounds, we evaluated an unexplored Nigerian medicinal plant—*Rutidea parviflora* DC. (family *Rubiaceae*) [17].

*R. parviflora* has been used for anti-inflammatory and anti-cancer activities among the indigenous communities in Delta state of Nigeria. The fruits are taken to induce vomiting and for the treatment of convulsions, epilepsy, spasm and paralysis [25]. However, there are no pharmacological and phytochemical studies reported. In this study, we report the extraction, isolation and identification of cytotoxic palmatine from *R. parviflora*, and its induction of apoptosis leading to ovarian cancer cell death.

## 2. Results

### 2.1. Bioassay-Guided Isolation and Identification of Palmatine

Both the organic and aqueous extracts of *R. parviflora* inhibited growth of the cultures with IC_50_ values of <10 μg/ml in OVCAR-4, OVCAR-8, A2780, and cisplatin resistant A2780 (A2780cis) ovarian cancer cell lines. Solvent partition of the organic extract yielded *n*-hexane, ethyl acetate, *n*-butanol and aqueous fractions. The *n*-butanol fraction showed the most potent cytotoxic effects followed by the ethyl acetate fraction (Table 1).

**Figure 1:**
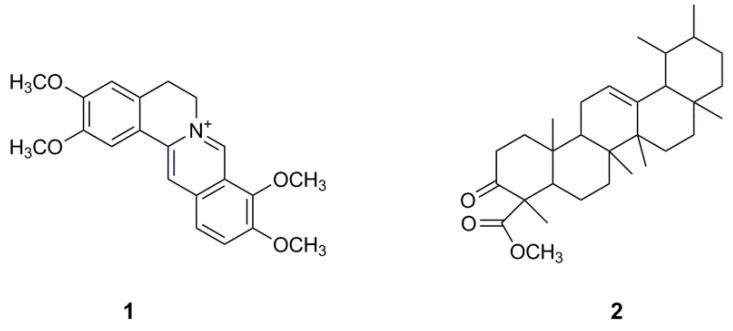
The chemical structure of palmatine (**1**) and urs-12-ene-24-oic acid, 3-oxo, methyl ester (**2**) isolated from *R. parviflora*.

Bioassay-guided fractionation and isolation of the bioactive compounds of *R. parviflora* was carried on the most potent *n*-butanol and ethyl acetate fractions. Nine sub-fractions were obtained from the column fractionation of the *n*-butanol fraction. HPLC purification of the most potent sub-fraction 3 was carried out to obtain a yellow powder, which was determined as palmatine (**1**) (Figure 1) by liquid chromatography coupled with mass spectrometry (LC-MS) (Figure S1), NMR spectroscopy and comparing with a standard palmatine.

Palmatine (**1**) showed potent cytotoxicity with IC_50_ (5.5–7.9 µM) in four ovarian cancer cell lines, but less cytotoxic to human ovarian epithelial (HOE) cell. The selectivity indexes (SI) of palmatine for cancer cells compared to HOE cells ranged from 3–5, while the SI of the clinically used carboplatin and paclitaxcel [24] only showed around 1–1.5 (Table 1). Further bioassay-guided fractionation of ethyl acetate fraction yielded urs-12-ene-24-oic acid, 3-oxo, methyl ester (**2**), which exhibited moderate inhibition of the growth of ovarian cancer cell cultures and also showed apparently mild effect on HOE cells (Table 1). Compound **2** has been previously identified by gas chromatography coupled with mass spectrometry (GC-MS) analysis from the ethanolic extract of *Canscora perfoliata* used in the treatment of poisonous bites [26].

### 2.2. Apoptosis Studies

#### 2.2.1. Caspase 3/7 Activity and Western Blotting Analysis

**Figure 2:**
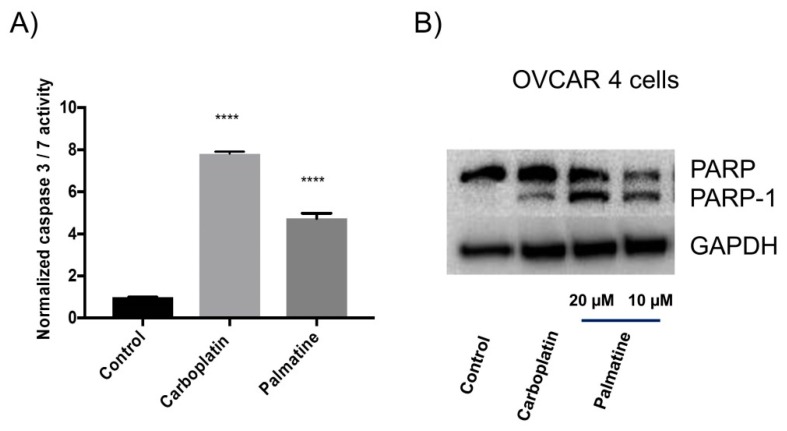
(**A**) The effect of carboplatin and palmatine (each 10 µM) on the caspase 3/7 activity at 48 h in OVCAR-4 cells. The caspase activity was measured and normalized with corresponding sulforhodamine B (SRB)-stained cells to estimate the surviving cell number. **** denotes that the result is significantly different (*p* < 0.001). The results were expressed as mean ±SEM, *n* = 3. (**B**) Detection of poly-ADP-ribose polymerase (PARP) cleavage by immunoblotting. OVCAR-4 cells were treated with palmatine (10 or 20 µM) and carboplatin (40 µM) for 48 h. The vehicle-treated cells served as the control.

In order to investigate the possible route of cell death caused by the compounds, the effect of palmatine on the activity of caspase 3/7 to evaluate apoptosis was determined in a selected OVCAR-4 cell line. It was derived from HGSOC tumor sample and regarded as one of the most suitable models of ovarian cancer [3]. Figure 2A demonstrated that palmatine as well as positive control-carboplatin significantly increased caspase 3/7 activity in comparison to the vehicle-treated cells for an experimental period of 48 h.

To confirm that the compounds induced apoptosis, caspases mediated cleavage of PARP was assessed by immuno-staining. As expected, significant PARP cleavage in OVCAR-4 cells was observed after treatment of palmatine and carboplatin (Figure 2B).

#### 2.2.2. Flow Cytometric Analysis

**Figure 3:**
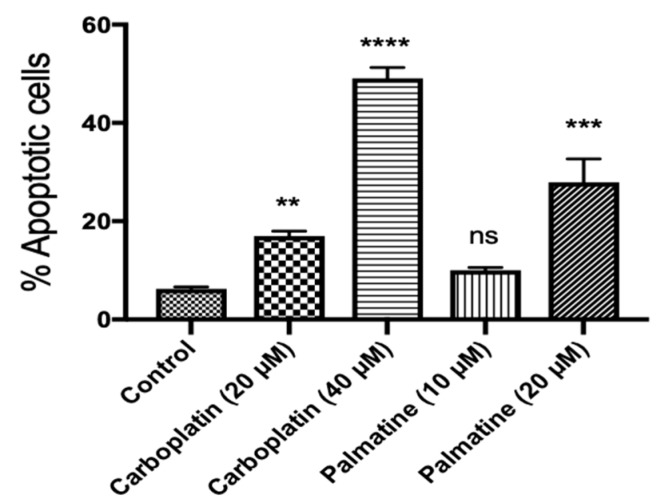
Flow cytometry analysis of the apoptotic effect of palmatine on OVCAR-4 cells after annexin V/PI staining. OVCAR-4 cells were treated with palmatine (10 or 20 µM) and carboplatin (20 or 40 µM) for 48 h. The vehicle-treated cells were used as the control. A representation of the quantification of the combined early and late phase apoptotic cells is shown. **, ***, and **** denote that the results are significantly different from the control with *p* < 0.01, 0.005, and 0.001, respectively. n.s. indicates *p* > 0.05. The results were expressed as mean ±SEM, *n* = 3.

Further investigation of the apoptosis inducted by palmatine (**1**) was carried out by means of annexin V/propidium iodide (PI) labelling, followed by flow cytometry analysis. Palmatine (**1**) induced concentration–dependent increase in the population of OVCAR-4 cells in the early and late stage of apoptosis compared to the control (Figure 3). The morphological changes of cells treated with palmatine were monitored microscopically at 48–72 h. The characteristic features of apoptosis such as blebbing and shrinkage of cells were also clearly observable by microscopy (Figure S2).

## 3. Discussion

The phytochemical and pharmacological characterization of *R. parviflora* was carried out in this study. Significant cytotoxic activities of both organic and aqueous extracts were demonstrated in ovarian cancer cells. Here we focused on the organic extract, however, different water-soluble and bioactive compounds could be discovered in the aqueous extract. From the organic extract, two cytotoxic compounds: palmatine (**1**), a quaternary protoberberine alkaloid, and urs-12-ene-24-oic-acid, 3-oxo, methyl ester (**2**), a triterpenoid, were isolated and identified for the first time. Palmatine showed significant inhibitory activity in the cell growth assay. Palmatine did show some preferential selective cytotoxicity for cancer cells, with slightly less potent inhibition of the growth of HOE cells (Table 1). The killing of cancer cells by the compound was likely to occur through induction of apoptosis, evidenced by significant increase in caspase 3/7 activity (Figure 2A), PARP cleavage (Figure 2B), annexin V/propidium iodide labelling of cells (Figure 3). PARP cleavage is a well-established method of demonstrating apoptosis as PARP is a substrate for caspase 3 and 7 and is cleaved in the course of apoptosis into two fragments [27,28]. PARP is a DNA repair nuclear enzyme which detects DNA fragmentation [29]. The cleavage of PARP-1 thus is a confirmation of apoptosis. This enzyme has been a validated drug target of developing successful PARP inhibitors for the treatment of ovarian cancer [10]. It is of interest to note that palmatine showed greater potency and selectivity compared to carboplatin. Importantly, it demonstrated an absence of cross-resistance in cisplatin-resistant A2780 cells (IC_50_ = 6.6 µM for A2780, and 5.5 µM for A2780cis cells). Because drug resistance still remains one of the main causes of failure for ovarian cancer treatment using platinum compounds [8].

Palmatine was previously found to be present in *Rhizoma coptidis*, an important medicinal plant commonly used in the traditional Chinese medicine [30] and the butanol fraction of *Phellodendron amurense* bark extract [31]. Our result is in agreement with the reported cytotoxicity of palmatine in prostate cancer cells [31] and breast cancer MCF-7 cell line [32]. In prostate cancer cells, the ribosomal protein S6, a downstream target of p70S6K and the Akt/mTOR signaling cascade was identified as a potential target of palmatine. Selective cytotoxicity of palmatine against prostate cancer cells was also reported [31]. Palmatine was shown to inhibit growth of pancreatic stellate cells (PSCs) and cancer cells alone or in combination with gemcitabine [33]. Such inhibition of growth and migration of pancreatic cancer cells was due to its suppression of glutamine-mediated changes in glioma-associated oncogene 1 (GL1) signalling, and induction of apoptosis [33]. Palmatine was found to be mainly located in endoplasmic reticulum and mitochondria of MCF-7 cells [33]. Further photodynamic treatment demonstrated photocytotoxicity of the naturally occurring photosensitizer palmatine in breast cancer MCF-7 [33] and colon adenocarcinoma HT-29 cells [34], causing significant cell apoptosis and increased intracellular reactive oxygen species levels. Palmatine has also been found to bind and stabilize parallel G-quadruplex DNA, indicating that it may be an inhibitor of telomere elongation and oncogene expression in humans [35]. Recently, palmatine from another traditional Chinese medicine *Mahonia bealei* was demonstrated to improve the survival of mice with colorectal cancer via the inhibition of inflammatory cytokines [36]. In the future work, it is necessary to test if palmatine behaves similar mechanism of action in ovarian cancer cells as those found in other cancers, and also to investigate its efficacy and safety in animal models of ovarian cancer.

## 4. Materials and Methods

### 4.1. Plant Material and Reagents

The stem bark of *R. parviflora* was collected in Delta state, Nigeria in February 2014. The plant was authenticated by A.O. Oziokowith of University of Benin, Nigeria. A voucher specimen (INTERCEDD/1588) was deposited in the herbarium at the International Centre for Ethnomedicine and Drug Development (INTERCEDD), Enugu state, Nigeria. Trichloroacetic acid (TCA) was purchased from Fisher Scientific (Loughborough, United Kingdom) Carboplatin, glacial acetic acid, N,O-bis(trimethylsily) trifluoroacetamide (BSTFA) with 1% chlorotrimethylsilane (TMCS), palmatine chloride, pyridine, sulforhodamine B (SRB) sodium salt, Trypsin-EDTA solution, and Trizma base were purchased from Sigma Aldrich (Gillingham, United Kingdom). Fetal bovine serum (FBS), penicillin-streptomycin, and RPMI 1640 medium were purchased from Lonza (Visp, Switzerland).

### 4.2. Extraction of Plant Materials

The plant materials were extracted according to the reported method [37]. The stem bark powder (1 kg) was macerated in a mixture of dichloromethane and methanol (1:1) for three times. The obtained residue was further macerated in methanol to yield the methanol extract. Both extracts were combined and evaporated to yield the total organic extract (7.2 g). Water was then added to the plant residue to obtain the aqueous extract (1.5 g) after freeze drying. The organic extract of *R. parviflora* was further partitioned in water using different organic solvents to obtain the *n*-hexane fraction (2.0 g), the ethyl acetate fraction (1.3 g), the *n*-butanol fraction (0.9 g) and the aqueous fraction (0.5 g).

### 4.3. Analysis of the Bioactive Fraction of R. parviflora by Gas Chromatography–Mass Spectrometry (GC–MS)

The bioactive fraction (1.0 mg) and isolated compound (**2**) of *R. parviflora* was incubated with 10 μl of pyridine and 50 μl of BSTFA (with 1% TMCS) in the oven at 37 °C for 2 h. The resulting trimethylsilyl (TMSi) derivatives were submitted to GC–MS analysis [16,38,39].

### 4.4. Liquid Chromatography Mass Spectrometry (LC-MS) Analysis

The isolated compounds were analysed by LC-MS, to determine their molecular mass, and their retention times compared with purchased standard compounds. The Agilent technologies 1260 Infinity coupled to 6530 Accurate mass Q-TOF LC-MS system was used (Agilent Technologies, Santa Clara, CA, USA). The gas temperature was 320 °C, with a dry gas flow rate of 11 L/min. Electrospray ionization (ESI) was operated at a voltage of 4000 V, an *m*/*z* range of 100–2000. The samples were injected at a volume of 5 µl and the run time was 15 min. The data was analysed by the Agilent mass-hunter qualitative analysis software.

### 4.5. NMR Spectroscopy

^1^H NMR spectra of the isolated compounds in CDCl_3_ or CD_3_OD were obtained with a Bruker 1D (DPX-300) NMR spectrometer for 300 MHz (Bruker, Billerica, MA, USA), and Bruker 1D (DPX-500) for 500 MHz. ^13^C NMR spectra were obtained at 125 MHz with a Bruker NMR spectrometer. ACD/Labs 10 Freeware (Advanced Chemistry Development Inc., Toronto, ON, Canada) was used in the Analysis of the NMR Spectra.

### 4.6. Purification and Isolation of Bioactive Compounds

The *n*-butanol fraction was submitted to silica gel column chromatography by eluting with ethyl acetate/methanol. Sub-fractions were subjected to high performance liquid chromatography (HPLC) on a semi-preparative column with a mobile phase composition of 0.1% trifluoroacetic acid (TFA) as solvent A and 80% acetonitrile with 0.1% TFA as solvent B. The gradient began with 100% of A for 5 min and increased to 80% B over 25 min. Then the gradient was increased to 100% B and maintained for 6 min. Palmatine (10 mg, **1**) was obtained as a yellow powder. LC positive ESI-MS, *m/z*: 352.1548 (Figure S1). ^1^H NMR (500 MHz, CD_3_OD), *δ* 9.76 (1H, s, H-8), 8.80 (1H, s, H-13), 8.12 (1H, d, J = 9.0 Hz, H-11), 8.01 (1H, d, J = 9.1 Hz, H-12), 7.67 (1H, s, H-1), 7.05 (1H, s, H-4), 4.92 (2H, t, J = 6.5 Hz, H-6), 4.21 (3H, s, OCH_3_), 4.11 (3H, s, OCH_3_), 3.99 (3H, s, OCH_3_), 3.94 (3H, s, OCH_3_), 3.28 (2H, t, J = 6.4 Hz, H-5). ^13^C NMR (125 MHz), *δ* 152.5, 150.5, 149.5, 145.0, 144.4, 138.5, 133.9, 128.7, 126.7, 123.0, 121.9, 119.9, 119.1, 110.8, 108.6, 61.1 (OCH_3_), 56.3 (OCH_3_), 55.9 (OCH_3_), 55.6 (CH_2_), 55.3 (OCH_3_), 26.4 (CH_2_). These data are consistent with those reported [40,41].

The ethyl acetate fraction (RP-EA) of *R. parviflora* was also fractionated on silica gel column with a solvent gradient range of 100% *n*-hexane to 100% ethyl acetate to yield ten sub-fractions. Sub-fraction F2 was treated with hot methanol and recrystallized at a temperature of 4 °C to yield a white crystal (yield: 12.8%), urs-12-ene-24-oic acid, 3-oxo, methyl ester (**2**), referring as EA2 (Figure 1), by GC-MS and ^1^H NMR. EI-MS, *m/z* (%): 468 [M]^+^ (2), 453 (1), 218.2 (100), 203.2 (15), 189.2 (21), 178.1 (6), 161.1 (10), 135.1 (18), 21.1 (17), 95.1(22), 81.1 (18), 69.1 (20), 43.1 (48). ^1^H NMR (300 MHz, CDCl_3_) δ: 5.27 (brs), 3.49 (s, 3H, O-CH_3_), 1.90-2.32 (m), 0.80-1.70 (m). Its ^1^H NMR is consistent with those reported [42].

### 4.7. Cell Culture

The human ovarian cancer cell lines, OVCAR-4, OVCAR-8, A2780, cisplatin-resistant A2780 (A2780cis) were bought from American Type Culture Collection (Manassas, VA, USA), and human ovarian epithelial (HOE) cells immortalized using SV40 large T antigen (Catalogue number: T1074) were purchased from Applied Biological Materials Inc. (Richmond, BC, Canada). All cells were cultured in Roswell Park Memorial Institute (RPMI) 1640 medium supplemented with 10% fetal bovine serum (FBS), penicillin-streptomycin (50 U/ml) and glutamine (2 mM).

### 4.8. Cell Growth Assay

The SRB assay was used to evaluate the effects of the plant extracts and pure compounds on the growth of ovarian cancer and HOE cell lines after treatment of 72 h [16,20,43]. 100 mg/ml concentrations of the plant extracts were prepared using dimethyl sulfoxide (DMSO) (organic extracts) and media (aqueous extracts) as stock solutions. 20 mM of purified compounds (**1** and **2**) and carboplatin were prepared in DMSO and used in the assay. The 0.1% DMSO in growth media was added to the cells; referred to as vehicle-treated cells (control). Nine concentrations of the drugs were prepared by using a two-fold serial dilution. Each well of the 96-well plates were seeded with 80 µl of the ovarian cancer and HOE cells at a density of 2000 cells/well, except for OVCAR-4 which was plated at a density of 5000 cells/well. After 24 h, 20 µl of plant extracts (1 mg/ml) after further 100-fold dilution of the stock solution in the medium or pure compounds were added and the cell cultures were kept in the incubator at 37 °C under 5% CO_2_ for 72 h in a humidified atmosphere. The medium was decanted and the cells fixed with 10% TCA on ice for 30 min and dried. The dried plates were stained with 0.4% SRB for 30 min, washed with 1% acetic acid and dried. 100 µL of Tris-base (10 mM) were added to the plates and shaken for 10 min to solubilise the protein-bound SRB dye. The absorbance at 570 nm was measured using a multi-mode microplate reader BioTEK Synergy 2 (Winooski, VT, USA). The recorded data was analysed by non-linear regression using the GraphPad PRISM software (GraphPad Software v6.0, San Diego, CA, USA) to fit a 4 parameter sigmoidal dose-response curve to determine IC_50_ values and the Hill coefficient. Based on the mean IC_50_s obtained, the selectivity index (SI) was calculated for each bioactive compound and carboplatin using the following formula:

(1) $$\mathrm{SI}\;=\;\frac{{\mathrm{IC}}_{50}\left(\mathrm{HOE}\;\mathrm{cell}\;\mathrm{line}\right)}{{\mathrm{IC}}_{50}\left(\mathrm{cancer}\;\mathrm{cell}\;\mathrm{line}\right)}$$

The SI value obtained is an indication of the preferential selectivity in the cytotoxicity of the compound for cancer cells. A large value suggests that the compound would be more cytotoxic to cancer cells than HOE cells.

### 4.9. Caspase 3/7 Activity

Caspase 3/7 activity was measured in cells pre-treated with vehicle (0.1% DMSO in growth medium), carboplatin, palmatine (**1**) and urs-12-ene-24-oic acid, 3-oxo, methyl ester (**2**) by use of the caspase 3/7 Glo-reagent obtained from Promega (Southampton, UK). For the determination of caspase 3/7 activity, OVCAR-4 cells were seeded in 96-well plates at a density of 5000 cells/well, and exposed to the compounds at different concentrations for 48 h. Then 25 µl of caspase 3/7 Glo-reagent was carefully added to the cells in the dark. The foiled plates were placed on a rocker for 30 min before measurement of luminescence.

### 4.10. Western Blotting

The measurement of PARP cleavage was carried out as described previously [20]. Briefly, six-wells plates seeded with OVCAR-4 cells at a density of 300,000/well and treated with 0.1% DMSO vehicle, 10 µM or 20 µM of palmatine or 40 µM of carboplatin for 48 h. The cells were collected, trypsinized, washed with cold phosphate buffered saline (PBS) and lysed with radioimmunoprecipitation assay (RIPA) buffer consisting of 20 mM Hepes, 150 mM NaCl, 2 mM EDTA, 0.05 mM pepstatin, 0.12 mM leupeptin, 1 mM phenylmethylsulfonyl fluoride, 0.5% sodium deoxycholate and 1% NP40.

The protein concentrations were evaluated by the bicinchoninic acid (BCA) Assay. About 10 µg protein of each cell lysate sample was carefully added to NuPAGE sample buffer made up with 5% *β*-mercaptoethanol, before electrophoresis using sodium dodecyl sulphate polyacrylamide gel electrophoresis (SDS-PAGE), for 15 min at a temperature of 70 °C to denature the proteins. The denatured proteins were added to a 4–20% Tris-Glycine polyacrylamide gradient gel to separate the proteins from the cell lysates using 100 mM Hepes, 100 mM Tris and SDS (1%) as running buffer.

The separated proteins were transferred to Amersham Hybond P (GE Healthcare Life Sciences, Buckinghamshire, UK) 0.45 µm polyvinylidene difluoride membrane and incubated in transfer buffer with a composition of 200 mM glycine, 25 mM Tris, 10% methanol and 0.075% SDS for 1.5 h. The membrane was again incubated in Tris buffered saline with tween (TBST, 150 mM NaCl, 0.1% Tween 20, 50 mM Tris hydrochloride, and 5% skimmed milk powder, pH 7.4) for 1.5 h on a rocker at room temperature to achieve blocking of the membranes. The membrane was subsequently incubated overnight at 4 °C in the buffer with primary antibody against poly-ADP-ribose polymerase (Catalogue number: 9542, Cell Signaling Technology Inc., London, UK) (1:1000) and antibody against glyceraldehde-3-phosphate dehydrogenase (GAPDH) (Catalogue number: MAB374, Millipore, Watford, UK) (1:5000). The membrane was washed several times with TBST, before incubation in the buffer with IgG secondary antibody conjugated with horseradish peroxidase (HRP) (Catalogue number: 7074, Cell Signaling Technology Inc.) (1:2000) for 1 h at room temperature on a rocker. Following several washes in TBST, the membrane was analyzed on a FluorChem M Imager (ProteinSimple, San Jose, CA, USA), for the visualization of the protein bands using the UptiLight HRP chemiluminescent substrate according to the manufacturer’s instructions (Uptima, San Jose, CA, USA).

### 4.11. Flow Cytometry

Flow cytometry analysis of annexin V/PI labelled treated cells were carried out as described as before [20,21]. Briefly, OVCAR-4 cells were seeded into 12-well plates and exposed to the treatment compounds at the indicated concentrations, followed by annexin V labelling of the cells using an annexin V-FITC kit procured from Miltenyi biotech (Bergisch Gladbach, Germany).

### 4.12. Statistical Analysis

The cytotoxicity, caspase activity and flow cytometry results were presented as mean values ± standard error of means (SEM). Statistical analysis was performed using one-way analysis of variance (ANOVA) and GraphPad Prism software v6.0 for the determination of statistical significance of difference between means. *p* < 0.05 were considered statistically significant.

## 5. Conclusions

In conclusion, this is the first report of the cytotoxic activities of *R. parviflora*, a medicinal plant, from folk medicine in Nigeria. Palmatine was isolated and identified from this unexplored plant, which provides a new source of palmatine. This study has also shown that palmatine possesses cytotoxicity, with apoptosis as the route of cell killing. Therefore, palmatine is a potential lead compound for the development of treatment of ovarian cancer, and merits further investigation.

## Figures and Tables

**Table 1:** Cytotoxicity (IC_50_) and selectivity index (SI) of the extracts, fractions, and isolated compounds of *R. parviflora* in ovarian cancer cell lines and an immortalized human ovarian epithelial (HOE) cell line after 72 h treatment. IC_50_ is the half maximal inhibitory concentration of extracts, fractions, or compounds. SI is a ratio of the measured IC_50_ value against HOE to the measured IC_50_ value against each cancer cell line. The results are expressed as mean ±SEM (*n* = 3). n.d., not determined.

Extract, Fraction and Compounds	OVCAR-4	OVCAR-8	A2780	A2780cis	HOE
	(µg/ml)				
Organic extract	6.6 ± 1.6	8.7 ± 0.5	3.2 ± 0.3	n.d.	n.d.
Aqueous extract	n.d.	5.9 ± 0.03	2.2 ± 0.5	3.7 ± 0.03	n.d.
*n*-Hexane fraction	23.3 ± 1.0	18.3 ± 0.3	7.3 ± 0.8	n.d.	n.d.
Ethyl acetate fraction	5.4 ± 0.3	5.8 ± 0.4	2.5 ± 0.2	n.d.	n.d.
*n*-Butanol fraction	2.6 ± 0.1	2.6 ± 0.3	1.7 ± 0.2	n.d.	n.d.
Aqueous fraction	22.9 ± 1.3	22.1 ± 1.1	12.8 ± 1.3	n.d.	n.d.
	**(µM)**				
Palmatine (**1**)	7.4 ± 0.3	7.9 ± 0.5	6.6 ± 0.5	5.5 ± 0.9	25.1 ± 5.0
SI for **1**	3.4	3.2	3.8	4.6	-
Urs-12-en-24-oic acid, 3-oxo-, methyl ester (**2**)	85.4 ± 2.4	48.9 ± 2.0	31.6 ± 3.3	n.d.	>200
SI for **2**	>2	>4	>6.5	-	-
Carboplatin	11.1 ± 0.4	10.8 ± 1.3	16.0 ± 1.0	>100	15.2 ± 3.0
SI for carboplatin	1.4	1.4	0.95	<0.15	-

## Supplementary Materials

The following are available online at https://www.mdpi.com/2072-6651/11/4/237/s1, Figure S1: LC-MS chromatogram and MS of palmatine isolated from *Rutidea parviflora*. Figure S2: The effects of palmatine (20 µM) on cell morphology of OVCAR-4 cells monitored by light microscope for 48–72 h.
