# Supplementary material for: Palmatine from Unexplored Rutidea parviflora Showed Cytotoxicity and Induction of Apoptosis in Human Ovarian Cancer Cells
Source: Toxins (Basel). 2019 Apr 25;11(4):237. doi: 10.3390/toxins11040237 (PMC6521182; doi:10.3390/toxins11040237)
Supplement: Supplementary file 1 [file toxins-11-00237-s001.pdf]

Supporting Information

Palmatine from unexplored *Rutidea parviflora* showed cytotoxicity and induction of apoptosis in human ovarian cancer cells

Okiemute Rosa Johnson-Ajinwo [1,2], Alan Richardson [1] and Wen-Wu Li [1,*]

1 Guy Hilton Research Centre, School of Pharmacy and Bioengineering, Keele University, Thornburrow Drive, ST4 7QB, Stoke-on-Trent, United Kingdom

2 Faculty of Pharmaceutical Sciences, University of Port Harcourt, Nigeria

*Corresponding author: w.li@keele.ac.uk

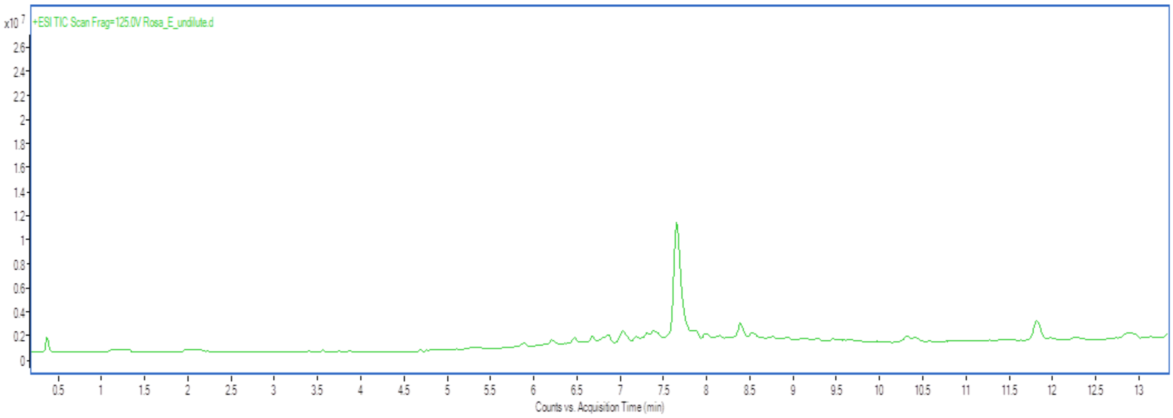

(**a**)

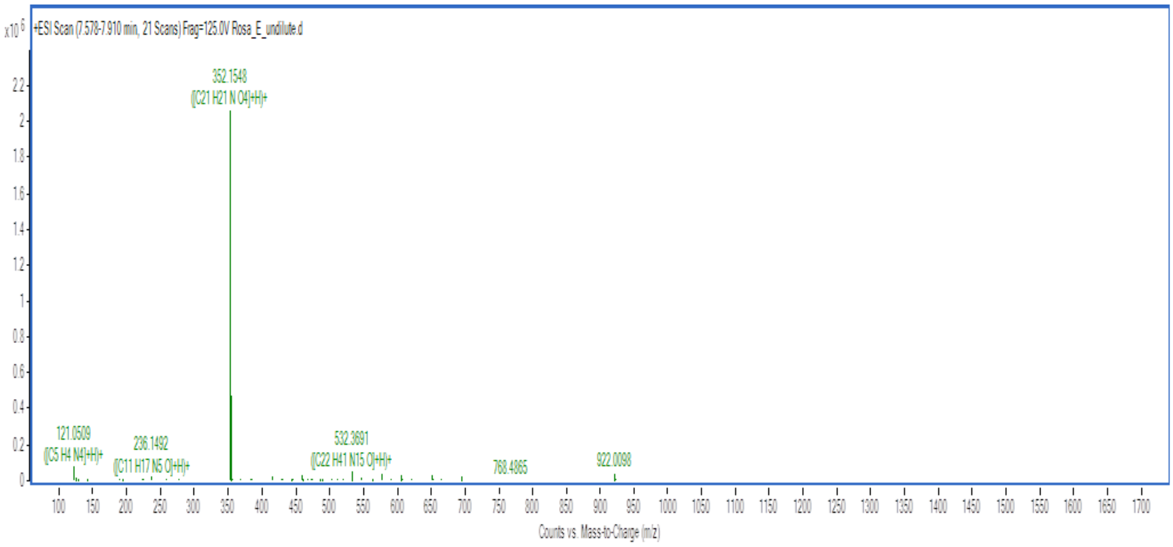

(**b**)

Figure S1. LC-MS chromatogram (**a**) and MS (**b**) of palmatine isolated from *Rutidea parviflora*.

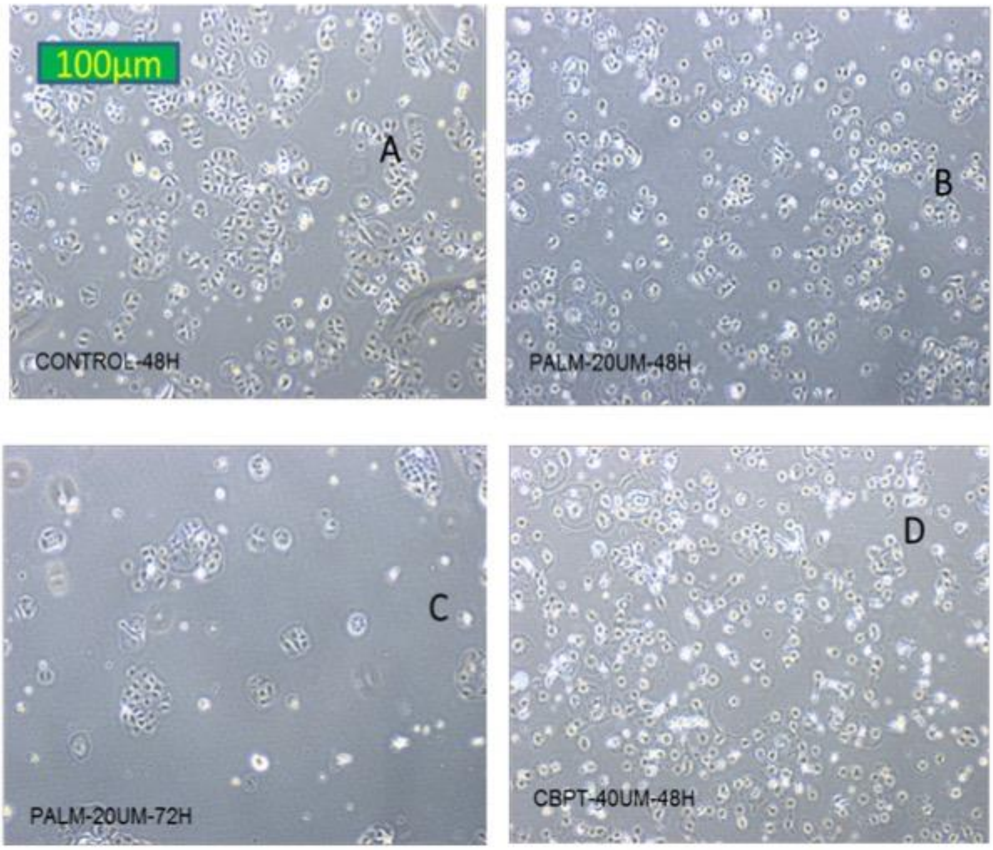

Figure S2. The effects of palmatine (20 μM) on cell morphology of OVCAR 4 cells monitored by light microscope for 48 or 72 h. The morphological changes observed in the vehicle-treated OVCAR 4 cells which were used as the control (**A**); OVCAR 4 cells treated with 20 μM palmatine at 48h (**B**); OVCAR 4 cells treated with 20 μM palmatine at 72h (**C**); and cells treated with 40 μM carboplatin at 48h (**D**). Representative images of three independent experiments were shown.
